# The Epidemiology, treatment, and complication of ameloblastoma in East-Indonesia: 6 years retrospective study

**DOI:** 10.4317/medoral.22185

**Published:** 2017-12-24

**Authors:** Muhammad Ruslin, Faqi N. Hendra, Arian Vojdani, David Hardjosantoso, Mohammad Gazali, Andi Tajrin, Jan Wolff, Tymour Forouzanfar

**Affiliations:** 1 Department of Oral and Maxillofacial Surgery Faculty of Dentistry Hasanuddin University, Makassar, Indonesia; 2 Department of Oral and Maxillofacial Surgery/Oral Pathology, VU University Medical Center/Academic Center for Dentistry Amsterdam (ACTA), Amsterdam, The Netherlands; 3 Department of Anatomy Faculty of Medicine Hasanuddin University, Makassar, Indonesia; 4 Department of Oral and Maxillofacial Surgery Undata General Hospital, Palu, Indonesia

## Abstract

**Background:**

Ameloblastoma is a neoplasm classified as a benign epithelial odontogenic tumor of the jaws, grow slowly and are locally invasive. The aim of the present study was to investigate the incidence, treatment, and complication of patients with ameloblastoma in East-Indonesia during six years retrospective study.

**Material and Methods:**

This retrospective study included 84 patients who were diagnosed with ameloblastoma from 2011 to 2016. There were 56 patients with treatment data available. Data from each patient, including gender, age, histologic type, the size of the tumor, radiologic form, tumor location, type of treatment, and complication were reviewed and analyzed retrospectively.

**Results:**

Fourteen patients were diagnosed with unicystic ameloblastoma (25%), thirty two patients with multicystic follicular ameloblastoma (57%) and ten patients with an unspecified multicystic ameloblastoma (18%). A total of about 35 patients were treated conservatively (62.5%) and 21 patients were treated radically (37.5%). Swelling was present as a pre-operative complication in all 56 cases (100%). There were no complaints concerning speech.

**Conclusions:**

The majority findings of the histologic type were multicystic ameloblastoma and their location were in the mandible. Most ameloblastoma were treated conservatively and reconstructions were made with only titanium plates and not bone graft.

** Key words:**Ameloblastoma, epidemiology, east Indonesia.

## Introduction

Ameloblastoma is a neoplasm classified as a benign epithelial odontogenic tumor of the jaws. Ameloblastomas grow slowly and are locally invasive. A vast majority of ameloblastomas are unilateral (95%) and occur in the posterior region of the jaws (85%). Most tumors are located in the mandible (80-93%) (1,2).

A systemic review by MacDonald-Jankowski *et al.* (3) showed that number of ameloblastomas per hospital was significantly higher in Asian or African populations than European or American hospitals. Lu *et al.* (4) studied the Chinese populations and showed a mean age of 31.4 years with a 1.5:1 male : female ratio and 90.8% of the tumors were in mandible. A study by Hatada *et al.* (5) on the Japanese population showed a mean age of 34.7 years with a 1.6:1 male : female ratio and 92.6% was located in the mandible. There was no study found in Indonesian population.

The main goals of ameloblastoma treatment are complete removal of the tumor and restoration of function and aesthetics (6). Broadly speaking, this can be achieved in two ways with surgical management; through conservative approach or radical approach (6-8). The conservative approach of treating ameloblastoma includes enucleation and curettage, whereas the radical approach includes resection or excision of a lesion that includes a measurable perimeter of investing bone (7).

The incidence of ameloblastoma, treatment, and complication has not been studied in the Indonesian population especially in East-Indonesia. The purpose of this study was to conduct a retrospective investigation to examine these important topics in East-Indonesia.

## Material and Methods

The data was collected for three months in Sulawesi, Indonesia during the period of April 13th-July 8th, 2016. The data was obtained from two hospitals, these were Hasanuddin University Dental Hospital in Makassar and Undata General Hospital in Palu. Patients’ files were collected for the period of January 2011-June 2016, where 84 patients were diagnosed with ameloblastoma.

The inclusion criteria of treatment data were diagnosed with ameloblastoma and treated for the same. The exclusion criteria were incomplete patients’ files (no treatment mentioned) and histopathological diagnoses other than ameloblastoma.

This study used a questionnaire to gather the data. Unknown data was left blank. Histologic type was confirmed by Pathology Anatomi (PA) result, if it was available in the medical files. The radiologic form was scored by one oral surgeon if radiographs were available.

Hong *et al.* made eight groups: anterior mandible (cuspid to cuspid); left and right posterior mandibles (premolar to molar); both rami (third molar to condyle); anterior maxilla (cuspid to cuspid); and both posterior maxilla (premolar to pterygoid plates) (9). In this study, the groups was used and altered four location: posterior maxilla; anterior maxilla; posterior mandible; anterior mandible. The cuspids in the maxilla and the mandible indicate the anterior border and posterior border. No difference was made between left and right.

Data from each patient, including gender, age, histologic type, location, the size of tumor, radiologic form, treatment of ameloblastoma, reconstruction, pre-operative, and post-operative complications were collected from medical reports and reviewed and analyzed retrospectively.

A database was created using Microsoft Excel and collected data was analyzed using SPSS v23 for statistical significance. Tests used were chi-square and an independent samples t-test. The significance level was <0.05.

## Results

Eighty-four patients were diagnosed with ameloblastoma between January 2011 and June 2016. Forty-nine patients were treated in Makassar and 35 in Palu. Eighty-four patients were used in epidemiological data in this study including 40 males (48%) and 44 females (52%). The treatment data was not available for all patients, files of 28 patients turned out to be unusable for this study, forty-five cases were obtained from Makassar and 11 from Palu totaling to 56 usable patient files for treatment, which included data of 21 males (37.5%) and 35 females (62.5%).

- Epidemiological Data

The mean age was 39.7 years (SD 17.4), with a minimum of five years and a maximum of 85 years. Out of 84 patients, 56 patients had a PA result included in the medical files. Fourteen patients were diagnosed with unicystic ameloblastoma (25%), thirty two patients with multicystic follicular ameloblastoma (57%) and ten patients with an unspecified multicystic ameloblastoma (18%). The location of tumor according to the four regions showed six cases in the maxilla, five (10.4%) in posterior and one (2.1%) in anterior, the mandible showed 38 (81.3%) cases in posterior and three (6.3%) cases in anterior. Radiographs were available for 56 patients. Nineteen radiolucencies (34%) were scored as uniloculated and 37 radiolucencies (66%) as multiloculated ( Table 1).

**Table 1:**
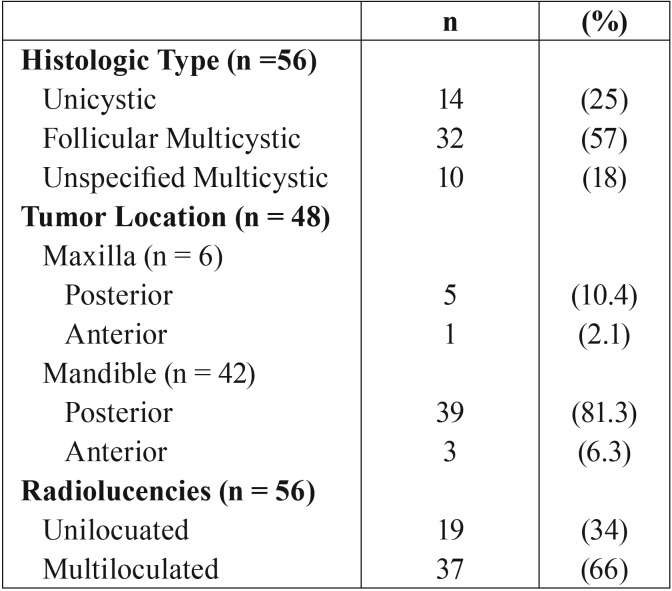
Disease-related results of patients with ameloblastoma.

- Treatment Data

Most patients were treated in 2014 but it is not known why there was such a spike in treatments in that year. The location of the tumor was known for 39 cases, three patients had a tumor in the maxilla. Of the 36 tumors in the mandible, ten tumors had no specified location, three were specified to be in the anterior region, and 23 were in the posterior region ( Table 2).

**Table 2:**
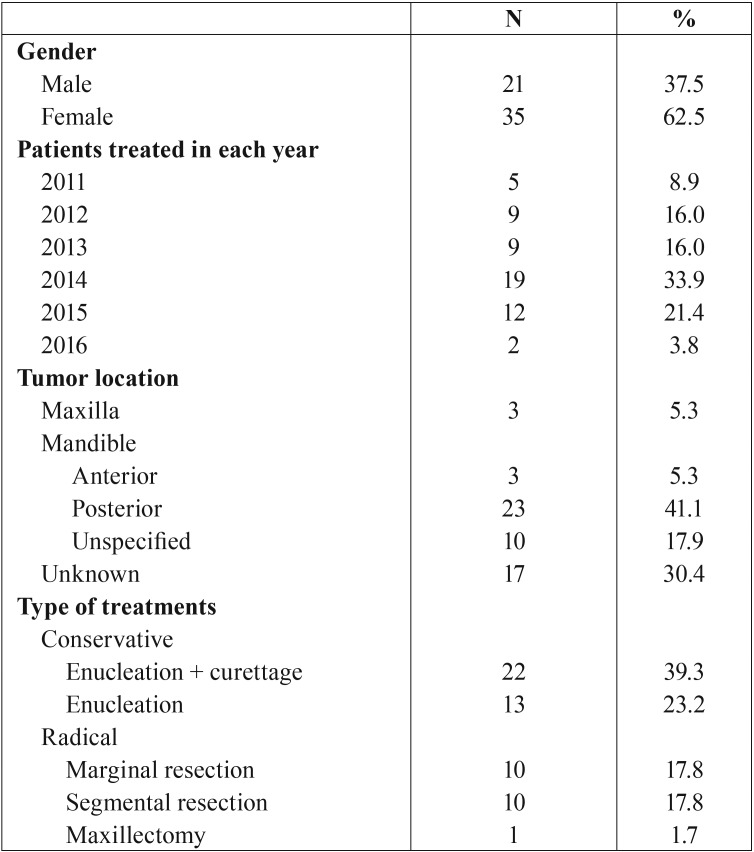
Gender distribution, number of patients treated in each year, location, and type of treatment.

A total of about 35 patients were treated conservatively (62.5%) and 21 patients were treated radically (37.5%). Most patients treated conservatively underwent enucleation and curettage (62.8%), the rest received only enucleation (37.25). Of the patients treated radically, about 10 patients received a marginal resection (47.6%) and 10 patients received segmental resection (47.6%), while only one patient underwent a maxillectomy (4.8%) (T Table 2).

A total of about five patients were documented to have received a reconstruction after tumor removal (8.9%). One of those reconstructions was an unspecified autogenous bone graft, the remaining four were reconstruction made with titanium plates. The patient with the bone graft had undergone a conservative treatment of enucleation and curettage. Three titanium plate reconstructions were performed after an enucleation.

The follow-up was documented for 56 patients (25%) for a periods of up to four years. Six recurrences were noted for these 56 patients (42.8%). Both of the patients who had undergone enucleation experienced a recurrence. Forty percent of the patients that had enucleation and curettage had a recurrence. One patient treated with segmental resection had a recurrence after four years ( Table 3).

**Table 3:**
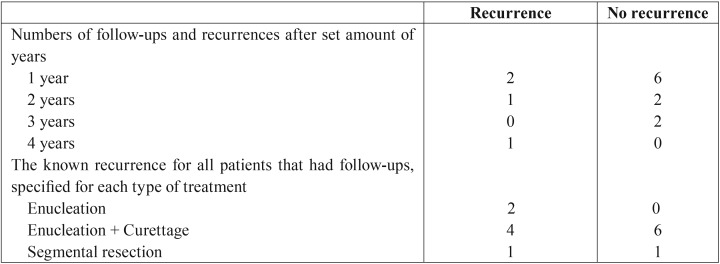
Follow-up and recurrences of patients with ameloblastoma.

- Complication

Swelling was present as a pre-operative complication in all 56 cases (100%). Out of 56 patients, the pain was present in eight cases (10%), numbness or an altered feeling was present in two cases (2%), breathing obstruction was present in one case (1%), and swallowing problems were present in two cases (2%). There were no complaints concerning speech ( Table 4).

**Table 4:**
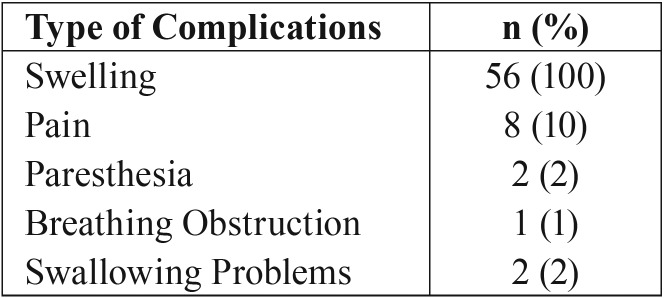
Pre-operative complications of patients with ameloblastoma. Some patients had more than one complication.

## Discussion

Patient files in Dental Hospital, especially in Makassar were barely maintained and documented a decade ago, but for the past five years, documentation has improved. This is a promising prospect for future (prospective research) in East-Indonesia. Setting up prospective studies for the treatment of ameloblastoma would most definitely help the continuing development and improvement of local health care.

Patients often wait for seeking medical care until their life is significantly impacted by the tumor (2). Since ameloblastoma is a slow growing tumor, it can take many years until a patient seeks medical care, at which point the treatment is much more complicated due to the size of the tumor. The patients in Indonesia showed a mean age of 39.7 years, which is similar to the main age of Caucasians (39.9 years) and Asian (41.2 years) according to Reichart *et al.* (10). In this study, the mean age was 41.00 for males and 38.64 for females. In the studies of Chukweneke *et al.* and Oomens *et al.* a higher age for males was also found (11,12).

Histologic distribution within this study was 25% unicystic ameloblastoma, about 57% multicystic ameloblastoma and an unspecified multicystic ameloblastoma 18%. These findings are similar to the findings from Gandhi *et al.* (13), which found 23% unicystic ameloblastoma and 77% multicystic ameloblastoma and findings from Saghravanian *et al.* (14), which found 24% with unicystic ameloblastoma, about 73% with multicystic ameloblastoma and 3% with extraosseous ameloblastoma.

Radiographically, the currents study had less uniloculated and more multiloculated radiolucencies compared to the finding of Gandhi *et al.* and Bansal *et al.* (13,15). It seems that the children have a higher percentage of uniloculated radiolucencies and a lower percentage of multiloculated radiolucencies, which is in accordance with a higher percentage of unicystic ameloblastoma and a lower percentage of multicystic ameloblastoma. But it should be stressed that both unicystic and multicystic ameloblastoma could show both uniloculated and multiloculated radiolucencies. In other words, the radiographic appearance is not dependent on the histological type (15-17).

The mean age of unicystic ameloblastoma (49.75 years) in the current study was higher than multicystic ameloblastoma (38.18 years). This difference was not significant. However, in literature, a lower age was found for unicystic ameloblastoma than multicystic ameloblastoma (1,14). Also, a higher percentage of unicystic ameloblastoma and a lower percentage of multicystic ameloblastoma were found within studies including only children, compared to studies including all ages (10,16,17).

Reconstructions were mostly done with titanium metal plates, which is notable in the modern literature that mainly discusses and offers studies about bone graft. Recent literature on titanium plates is mostly limited to case report (18,19). Older study show high rates of complications (20-22), which seems to be confirmed by this study where two out of four patients experienced post-operative complications; one patient had excessive wound bleeding and one patient experienced plate rejection after difficult closure during the surgery. The ameloblastoma reconstructions are less invasive and less expensive for the patient since no bone has to be grafted, which could explain why it is used so often East-Indonesia.

In East-Indonesia, most patients were treated conservatively (62.5%) despite a majority of patients being diagnosed with multicystic ameloblastoma. There is no explanation for this, but it could be that treatment is decided by the size of the tumor and not by the histological type. Patients may also deny radical treatment due to financial factors or reluctance towards the risk of deformities, lip numbness, malocclusion, or poor mastication (23).

The swelling was found in 70% cases in the study of MacDonald-Janskowski *et al.* (3). In this study, the swelling is the chief complaint. These swellings were larger in size compared with literature and the patients waited longer before seeking medical assistance.

A cultural reason would be that Indonesian people have a higher threshold of seeking medical assistance. The geographical restriction could also apply. On the Sulawesi Island, there are only a few big cities with hospitals and oral surgeons. The infrastructure and distances from their homes to these big cities could be a restricting factor to seek medical assistance.

The present study has several shortcomings. This study was limited to East-Indonesia (Makassar and Palu). Further study related to ameloblastoma is still required in other health centers in Indonesia. However, the number of treated patients is not equally distributed among the hospitals. Most patients are treated at the Dental Hospital Hasanuddin University Makassar and General Hospital Palu. Therefore, our results may not be generalizable to the whole population of Indonesia. Because this study was retrospective, the analysis may include an information bias. However, the results presented in this study are similar to the reports from other studies. Furthermore, the analysis in this report provides important data for improving the treatment plans for ameloblastoma surgery.

In the Indonesian retrospective study regarding ameloblastoma, the majority findings of the histologic type were multicystic ameloblastoma and their location was in the mandible. Most ameloblastomas were treated conservatively in East-Indonesia and reconstructions were mostly made with only titanium plates and not by bone graft, which is an older technique not used much in the Western world anymore. These reconstructions sometimes have complications that require more surgery or a longer hospital day.
